# A new method to optimize root order classification based on the diameter interval of fine root

**DOI:** 10.1038/s41598-018-21248-6

**Published:** 2018-02-13

**Authors:** Ying Liu, Guoliang Wang, Kunxia Yu, Peng Li, Lie Xiao, Guobin Liu

**Affiliations:** 1 State Key Laboratory of Eco-hydraulics in Northwest Arid Region of China, Xi’an University of Technology, Xi’an, 710048 China; 2 State Key Laboratory of Soil Erosion and Dryland Farming on the Loess Plateau, Institute of Soil and Water Conservation, Northwest A&F University, Yangling, 712100 China

## Abstract

Plant roots are a highly heterogeneous and hierarchical system. Although the root-order method is superior to the root diameter method for revealing differences in the morphology and physiology of fine roots, its complex partitioning limits its application. Whether root order can be determined by partitioning the main root based on its diameter remains uncertain. Four methods were employed for studying the morphological characteristics of seedling roots of two *Pinus* species in a natural and nitrogen-enriched environment. The intrinsic relationships among categories of roots by root order and diameter were systematically compared to explore the possibility of using the latter to describe root morphology. The normal transformation method proved superior to the other three in that the diameter intervals corresponded most closely (at least 68.3%) to the morphological characteristics. The applied methods clearly distinguished the results from the natural and nitrogen-rich environments. Considering both root diameter and order simplified the classification of fine roots, and improved the estimation of root lifespan and the data integrity of field collection, but failed to partition all roots into uniform diameter intervals.

## Introduction

Plant roots are highly heterogeneous and hierarchical. Roots that differ in diameter and order also differ in morphology, anatomy, and physiology^1^. Classifying roots by diameter is the most common research method due to its easy application^2–7^. Many studies define fine roots as those smaller than 1–2 mm in diameter^8,9^, whereas other studies define the first two or three root orders as fine roots^10,11^. However, recent research on root anatomy indicates that unlignified fine roots are mainly first- or second-order roots with a small number of third-order roots^12^, whereas third-order roots and beyond are mostly lignified. For tree species, most third-order roots have a mean diameter of less than 0.50 mm; for example, third-order roots of *Fraxinus mandschurica* Rupr. average 0.43 mm in diameter^13^, whereas those of *Acer saccharum* Marsh., *Populus euphratica* Olivier, *Quercus fabri* Hance, and *Pinus resinosa* Ait. are all smaller than 0.40 mm^10^. Thus, distinguishing fine and coarse roots by their diameter fails to reveal the differences in internal structure and function (i.e., order and position) of fine roots, resulting in large errors in estimating their turnover rates, lifespans^14,15^, and below-ground contributions^16^.

Studies on root system hierarchy show that the anatomy and physiology of roots change with higher root order. For example, diameter increases whereas specific root length (SRL) and specific root surface area (SRA) decrease with root order^10,17–20^; the main function changes from absorbing water and nutrients to transporting and anchoring^21–23^; the anatomical structure changes from primary to secondary^24^; the extent of mycorrhizal colonization^13^ and respiration intensity^17^ decreases; and, the lifespan is extended and the turnover rate declines^25,26^. Conversely, these parameters are more consistent among fine roots of the same order^14,23^. Therefore, lifespans and rates of turnover and respiration based on root order are significantly more reliable^10,11,27^. However, the root-order method also poses a problem. In a large field sample of root fragments or individual roots, it is difficult to identify the order of a given root or fragment. An easier method in which roots are grouped based on their diameter and order will overcome this drawback when classifying fine roots (smaller than 1–2 mm).

Intrinsic correlations do exist between root diameter and other root characteristics; for example, the diameter of fine roots tends to increase with root order. Several studies^28,29^ have shown an association between root order and diameter classification. Specifically, when a given fine root diameter interval encompasses most roots of a given root order, which match in number, length, and biomass, then studies on fine root lifespan and turnover rate will be less prone to errors^11^. Chang and Guo^30^ studied variations in the diameter of the first five root orders of 45 common tree species and showed that mean diameter increased exponentially with root order. Such studies thus indicate the possibility of establishing a relationship between fine root order and diameter.

Although the diameter ranges of different root orders overlap to some extent, tree species have numerous fine roots in the first several orders. The overlap between two adjacent orders of root diameters can be partitioned using mathematical methods such that most roots–whether coarse or fine–in the partitioned diameter range belong to the lower root order, whereas fine roots in another diameter range belong to the higher root order. In the present study, we assumed that root distribution in terms of diameter in every root order was normal or quasi-normal. According to the rule of normal distribution, namely the 68.3–95.0–99.7^31^ or three-sigma rule^31–33^, if the morphological parameters (e.g., length, area, and number of root tips) of 68.3% of roots in a given root order fall within the partitioned diameter interval, then that interval represents that particular root order. In accordance with the above assumption–namely that a diameter interval represents a root order – we partitioned the overlapping zone between two adjacent root orders using four methods. We then compared the results to determine the optimal partitioning method, and tested the chosen method by studying the roots of *Pinus tabuliformis* Carrière and *Pinus bungeana* Zucc. in a natural environment and those of *P*. *tabuliformis* in a nitrogen-enriched environment. This new method can simplify the classification of fine roots, more accurately estimate root lifespan, and improve data integrity of collected root fragments.

## Materials and Methods

### Root excavation

Pine seedlings were collected from a tree nursery in Xinjiazhai (33°40′N, 107°38′E) in Zhouzhi county in Shaanxi Province, China. This area has a temperate continental monsoon climate with an annual average temperature of 13.2 °C, precipitation of 674.3 mm, sunshine duration of 1993.7 h, and frost-free season of 225 d. Eighteen 2-year-old seedlings of *P*. *tabuliformis* (height 13.3–15.3 cm and collar diameter of 2.0–3.1 mm) and of *P*. *bungeana* (height 10.6–12.3 cm and collar diameter of 1.9–2.6 mm) were selected from the tree nursery in June 2010. A soil block containing the whole plant was dug around the plastic pots (each 5.0 cm in diameter and 25.0 cm tall) in which the seedlings had been placed at the time of transplanting. When the roots outgrew the feeding block, the entire seedling along with the block and surrounding soil of the root bed was dug out and packed into a plastic bag. All transplanted seedlings were transferred to the laboratory in a cold storage box maintained at 1–3 °C and then stored at −20 °C.

Another set of *P*. *tabuliformis* seedlings was transplanted into PVC tubes (35.0 cm in diameter and 40.0 cm tall) filled with forest soil in June 2010. The soil was a calcic cambisol^34^ consisting of aeolian loess highly prone to erosion. The physicochemical characteristics of the soil were as follows: density, 1.14 g/cm³; pH, 7.9 ± 0.2; total P, 1.40 ± 0.38 g/kg; total N, 0.73 ± 0.21 g/kg; and organic matter, 9.6 ± 0.75 g/kg (mean ± SD, n = 6). The tubes were given a 5.6 g/m² dose of N as urea (Fumin Agriculture Product Company, Xian, China) dissolved in 10 mL of distilled water. The dose was given on three separate occasions on days on which it rained (5 June and 18 September 2010 and 28 March 2011). The roots of these seedlings were excavated on 5 June 2011 following the same method described above. At that time, the seedlings were 14.1–16.4 cm tall and collar diameter ranged from 2.3 to 3.7 mm.

### Root dissection and scanning by the root-order method

The soil around the roots was washed off with cold deionized water (2–3 °C). The cleaned roots were then placed in culture dishes containing deionized water ice and sorted by order. The roots of each seedling were first divided into several segments based on Pregitzer’s root-order classification method^10^. Distal roots made up the first-order roots (order I), the next segment comprised order II roots, and so on, moving down the root system to order VI, which formed the largest category. Each root was removed using tweezers. The first-order roots were placed in a sink filled with iced water and immediately scanned (Expression 4490, Epson, Beijing, China); the other roots were placed on transparent films and then scanned (resolution of 300 dpi). Only live roots were measured; dead roots were discarded. Because only a few *P*. *tabuliformis* seedlings had roots up to order VI and all *P*. *bungeana* seedlings had roots only in the first five orders, only these orders were included for analysis. Roots from broken segments, which accounted for less than 1% of the total biomass of all five orders, were also excluded from analysis. Root length, diameter, and surface area were measured using a WinRHIZO 2010 image analyzer (Regent Instruments Inc., Ville de Québec, Canada). All roots were dried to a constant weight in an oven at 65 °C and then weighed with a balance scale to the nearest 0.001 g (Shanghai Precision Instrument Co., Ltd., Shanghai, China). Specific root lengths were calculated as the ratio of root length to root biomass (dry weight), and specific root surface areas as the ratio of root surface area to biomass (dry weight) for each root order or each root class by diameter.

### Diameter classification method

The diameter of every single root was calculated from the scanned images. Each root had its unique diameter, length, and surface area. To compare our results with those from earlier studies, we used the most commonly applied diameter thresholds and divided the data accordingly at intervals of 0.5 mm: Class D1 comprised roots with diameters <0.5 mm; D2, 0.5–1.0 mm; D3, 1.0–1.5 mm; D4, 1.5–2.0 mm; and D5, >2.0 mm.

### Correlation between root diameter and root order

The roots of *P*. *tabuliformis* (2,846 roots) were grouped by diameter using each of the four methods. To ascertain the correlation between these groups and root orders, roots of seedlings from *P*. *bungeana* (2,457 roots) and *P*. *tabuliformis* grown in nitrogen-enriched soil (1,244 roots) were also sorted and grouped by diameter. The four methods used were the probability distribution function interval, probability distribution function intersection, quartile averaging, and normal distribution transformation. Table 1 defines and provides the specific procedures of the four approaches.

**Table 1:** Methods of diameter interval division to represent morphological characteristics of root order.

Method name	Definition and description	Specific procedure
Probability distribution function interval	Analyses the probability distribution of two adjacent root orders diameters and determines an interval of diameter distribution (μ − σ, μ + σ) as per the 68.3-95.4-99.7 (or three-sigma) rule^31–33^, which corresponds to the main diameter intervals of root orders.	1) Determine distribution function that two adjacent orders of root diameters, and record parameters of the function.
		2) Calculate from the probability distribution plot a corresponding diameter interval within the interval marked by μ − σ and μ + σ of the distribution function for two adjacent orders of root diameters (μ is mathematical expectations and σ is standard deviation).
		3) Partition arithmetic mean of the upper limit of the lower-order root diameter and lower limit of the higher-order root diameter into ranges of two adjacent orders of root diameters, and determine the corresponding diameter interval for every root order in turn.
Probability distribution function intersection	Analyses the probability distribution functions of two adjacent orders of root diameters, with the diameter corresponding to the intersection point of both functions taken as the threshold for determining both root orders.	1) Determine distribution function that two adjacent orders of root diameters follow.
		2) Calculate from the probability distribution plot an intersection point of distribution functions for two adjacent orders of root diameters, with corresponding diameter taken as the threshold for determining both root orders.
		3) Determine corresponding diameter interval of every root order in turn.
Quartile averaging method	Measures the diameters of each order of roots, takes 25% and 75% quartiles as upper and lower limits of this order of root diameters, respectively, and calculates arithmetic mean of upper limit of the lower order of root diameter and lower limit of the higher order of root diameter of two adjacent root orders, taking that as a threshold value of two adjacent orders of root diameters.	1) Determine frequency distribution of diameters of each root order and identify diameters corresponding to 25% and 75% quartiles, respectively.
		2) Partition arithmetic mean of upper limit of the lower order of root diameter and lower limit of the higher order of root diameter into diameter ranges of two adjacent root orders and determine diameter interval corresponding to each root order in turn.
Normal distribution transformation	Converts diameter distribution data for every root order into normal distribution. According to probability density function of normal distribution and its characteristics, if a large number of statistically independent random variables exhibit a probability distribution similar to normal distribution, then, although the value range of a normal variable is from −∞ to +∞, 99.7% of the values will fall within μ − 3σ and μ + 3σ; 95.4% of the values will fall within μ − 2σ and μ + 2σ, and 68.3% of the values will fall within μ − σ and μ + σ, i.e., the 68.3–95.4–99.7 or three-sigma rule. For random variable *X*_*i*_, if function *Y*_*i*_ = *f(X*_*i*_*)* follows normal distribution *N(μ*, *σ)*, *σ*^2^ > 0, then *X*_*i*_ will follow normal distribution *Y*_*i*_ with parameters *μ* and *σ*^*2*^. Density function of function *Y*_*i*_ is:$$f(Yi)=\{\begin{array}{c}\frac{1}{Yi{\sigma }\sqrt{2\pi }}\exp [-\frac{Y{i}^{2}}{2}]\\ 0,\,X\le 0\end{array}(1)$$In the present study, it was assumed that various orders of root diameters of *P*. *tabuliformis* seedlings were statistically independent random variables and their distribution approximated normal probability distribution or can do so after data transformation. This assumption was valid if the diameter distribution of a root order satisfied 68.3% of the interval range, representing the main distribution range of this order of root diameters; that is, if 68.3% of the interval range corresponds to quartiles 15.85% and 84.15% and the corresponding *X*_*i*_ values of both quartiles are the threshold values capable of representing the main distribution range of this order of root diameter.	1) Obtain diameter data on every order of roots by means of root order classification and run the Kolmogorov–Smirnov test to check if every order of root diameter follows a normal distribution.
		2) If every order of root diameter follows normal distribution, directly calculate the diameter interval corresponding to the 68.3% quartile.
		3) If an order of root diameter does not follow normal distribution, conduct Johnson transformation such that each order of root diameter exhibits normal distribution, and then calculate the diameter interval corresponding to the 68.3% quartile.
		4) Partition arithmetic mean of upper limit of the lower order of root diameter and lower limit of the higher order of root diameter into diameter ranges of two adjacent root orders, and then determine diameter interval corresponding to each root order in turn.

### Comparison of four methods

The present study assumed that the four partitioning methods were only valid when at least 68.3% of the different fine root parameter values fell within the partitioned root diameter intervals and corresponded to the morphological characteristics of a given root order. When this criterion was adhered to, we judged the diameter interval as capable of approximately expressing the morphological characteristics of the corresponding root order.

We maintained that if the different methods satisfied the above condition, it was more convenient to use the method that directly classified raw statistical data and necessitated fewer transformation steps.

### Data analysis

Analysis of variance (ANOVA) was used for ascertaining if the differences between root parameters varying with order and diameter were significant (α = 0.05), and the least significant difference (LSD) test was used to determine whether the effects of the classification method based on root order or diameter for each parameter were significant (α = 0.05). All graphs were drawn using SigmaPlot ver. 10.0 and Minitab ver. 16.0, and ANOVA was conducted using SPSS ver. 21.0.

## Results and Discussion

### Root morphology described by root order and diameter classification

**Figure 1:**
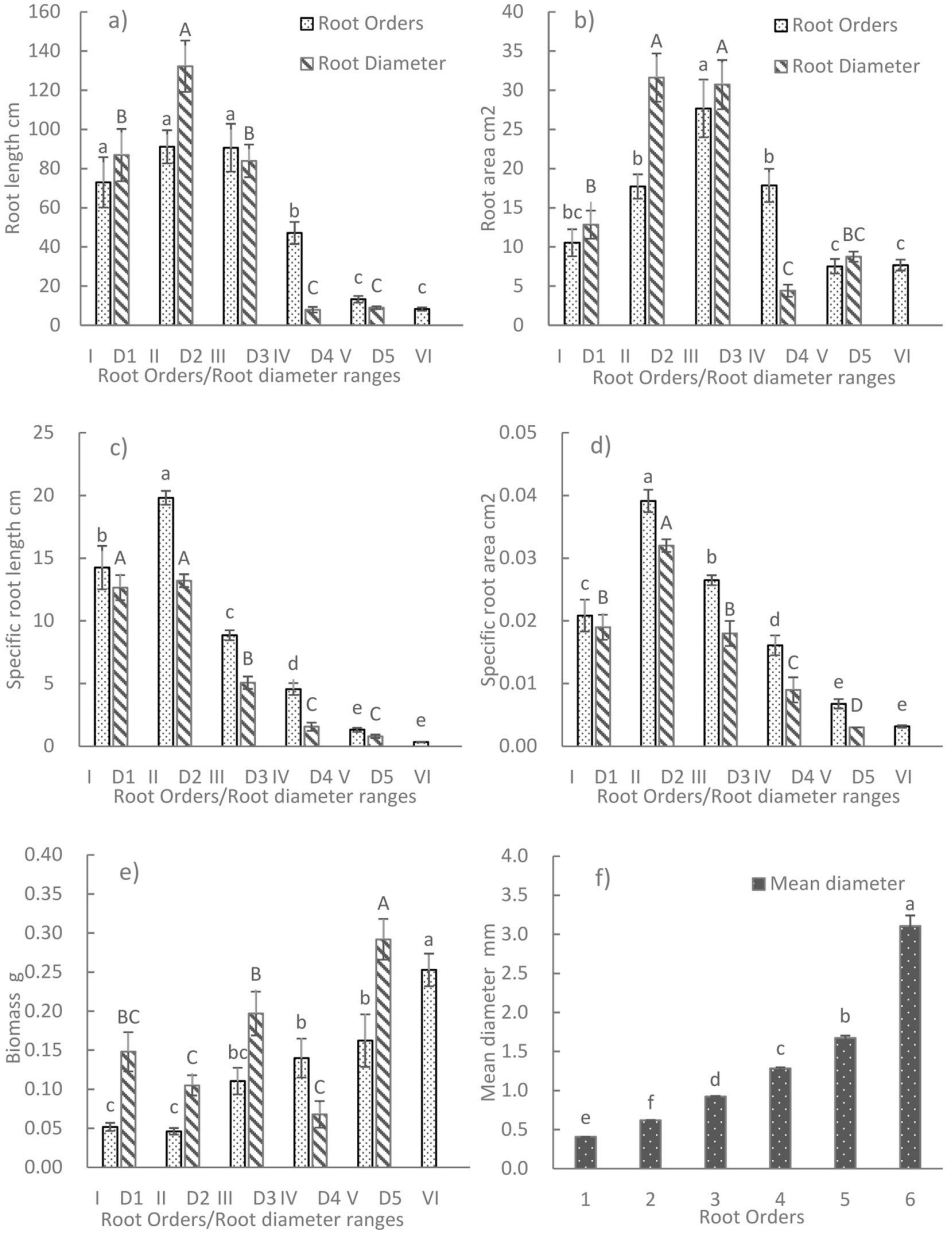
I to VI correspond to root orders 1 to 6; D1 to D5 are five groups based on root diameter: D1, ≤0.5 mm; D2, 0.5–≤1.0 mm; D3, 1.0–≤1.5 mm; D4, 1.5–≤2 mm; D5, >2 mm; different lowercase letters indicate significant differences among different orders at the 0.05% level; different capital letters indicate significant differences among different root diameter ranges at the 0.05% level; values are mean ± SE, n = 18 seedlings.

The 2-year-old *P*. *tabuliformis* seedlings had up to six root orders. As the root order of the *Pinus* seedlings increased, root diameter and biomass also increased significantly, whereas the other morphological parameters decreased (Fig. 1). Root length (RL), root area (RA), specific root length (SRL), and specific root surface area (SRA) in the first three root orders were much higher than those in the other orders and accounted for 78.1–94.2% of total RL and 58.4–75.3% of total RA. Similarly, SRL and SRA in the first three root orders were 7–22 times and 1.5–5 times higher, respectively, than that in the other orders.

The diameter and root order classification results were similar: the morphological parameters increased with the increase in root diameter; RL and RA in the 0–1.5 mm diameter class accounted for 93.8% and 84.4% of the respective totals; those in the 1.5–2.0 mm and >2.0 mm categories accounted for 3.7% and 2.6% of total RL and 6.9% and 8.8% of total RA, respectively; both SRL and SRA showed the same pattern: SRL in the <1.5 mm category was 3–7 times and SRA was 1.5–3 times that in the other ranges. The two classification methods were similar in describing root morphology; for example, both RL and RA decreased as the root order (or diameter) increased and most SRL and SRA values were accounted for by the lower root orders and diameters. This correlation showed that the two methods provided similar results, confirming the findings of Chang and Guo^30^.

### Partitioning diameter interval

#### Probability distribution function interval

**Figure 2:**
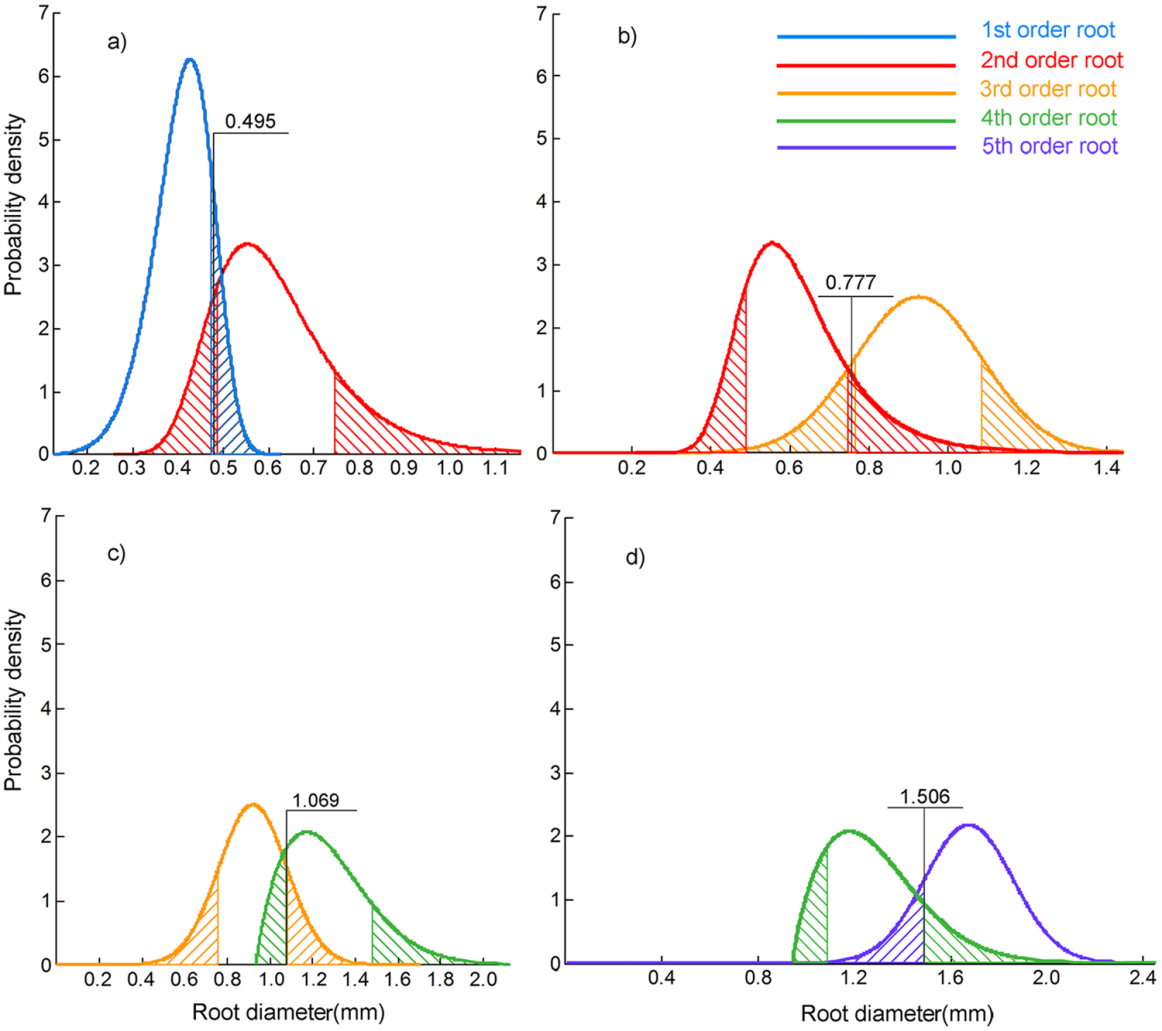
Solid lines in different colours represent probability distribution functions constituted by diameters corresponding to the 1st–5th orders of roots, respectively; (**a**) probability distribution functions of diameter for first and second orders of roots, where blue hatched area is the diameter interval to which the right tail corresponds when distribution probability of first-order roots is 68.3%; red hatched area is the diameter interval to which two tails correspond when distribution probability of second-order roots is 68.3%; and arithmetic mean for hatched overlapping area is the diameter interval threshold of first-order and second-order roots, namely 0.495 mm. (**b**) Red hatched area is the diameter interval to which the tow tails corresponds when distribution probability of second-order roots is 68.3%; orange hatched area is the diameter interval to which two tails correspond when distribution probability of third-order roots is 68.3%; and arithmetic mean for hatched overlapping area is the diameter interval threshold of second-order and third-order roots, namely 0.777 mm. (**c**) Orange hatched area is the diameter interval to which the tow tails corresponds when distribution probability of third-order roots is 68.3%; green hatched area is the diameter interval to which two tails correspond when distribution probability of fourth-order roots is 68.3%; and arithmetic mean for hatched overlapping area is the diameter interval threshold of third-order and fourth-order roots, namely 1.069 mm. (**d**) Green hatched area is the diameter interval to which the tow tails corresponds when distribution probability of fourth-order roots is 68.3%; purple hatched area is the diameter interval to which the left tails correspond when distribution probability of fifth-order roots is 68.3%; and arithmetic mean for hatched overlapping area is the diameter interval threshold of fourth-order and fifth-order roots, namely 1.506 mm.

The diameter interval partitioning results obtained by the probability distribution function interval method are shown in Fig. 2. Across all first-order roots, as judged by diameter, the 68.3% probability corresponded to the 0.350–0.469-mm diameter interval; for second-order roots, 68.3% corresponded to the 0.521–0.732-mm diameter interval. The diameter ranges for these two root orders overlapped within the 0.469–0.521-mm interval. The arithmetic mean of the upper and lower limits of the diameter range, 0.495 mm, was set as the partitioning threshold for separating first-order and second-order roots. According to this method, the threshold intervals of the second-third-fourth-fifth order roots were 0.777 mm, 1.069 mm, and 1.506 mm.

#### Probability distribution function intersection

**Figure 3:**
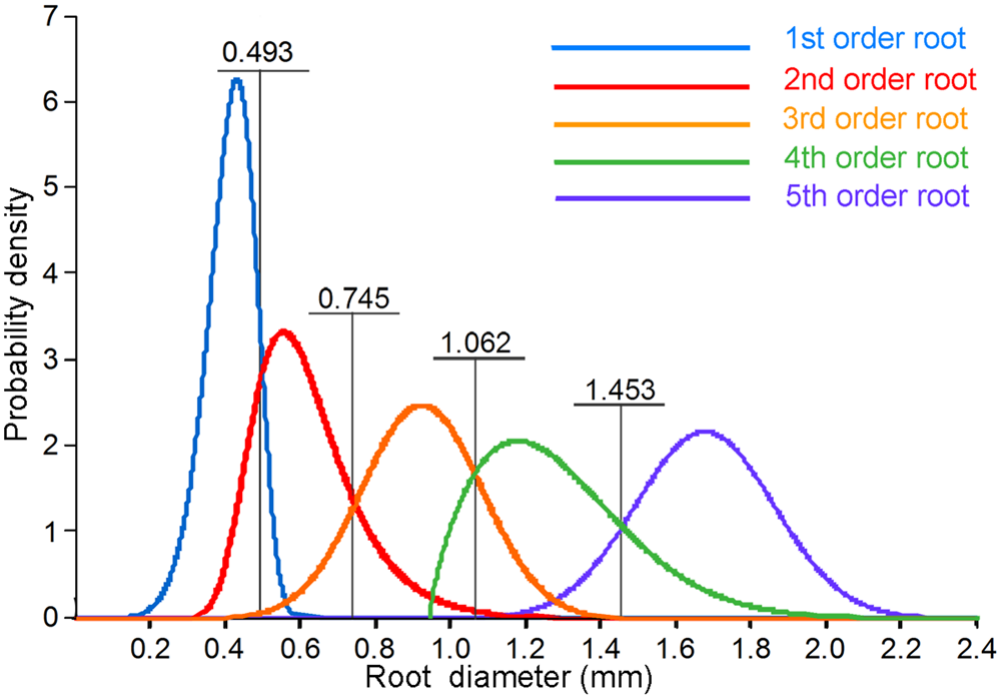
Solid lines in different colours represent probability distribution functions constituted by diameters corresponding to the 1st–5th orders of roots, respectively; intersection points of probability function distribution curves of diameter for two adjacent root orders are simply diameter interval threshold values.

With the diameters corresponding to the intersection points of the probability distribution functions for the five orders of roots (Fig. 3) deemed as the partitioning threshold values of the adjacent next-order roots, the diameter threshold values of the first to fifth orders were 0.478 mm, 0.732 mm, 1.062 mm, 1.453 mm, and >1.453 mm, respectively.

#### Quartile averaging

The threshold values obtained from partitioning by quartile averaging were 0.486 mm, 0.768 mm, 1.088 mm, and 1.484 mm, and the percentages of the first to fifth orders within the diameter ranges partitioned according to the above threshold values were 87.9% for first-order roots with diameters ≤0.486 mm, 68.6% for second-order roots in the 0.486–0.768-mm group, 67.2% for third-order roots in the 0.768–1.088-mm group, 67.5% for fourth-order roots in the 1.088–1.484-mm group, and 77.5% for fifth-order roots in the >1.484-mm group.

#### Normal transformation

Analysis of the data transformed with the Johnson function (Table 2) showed that diameters of first-order roots varied between 0.169 mm and 0.545 mm and those of second-order roots varied between 0.382 mm and 1.357 mm, thus showing considerable overlap. However, 68.3% of first-order roots were within the 0.344–0.474-mm diameter range and 68.3% of second-order roots were within the 0.484–0.753-mm diameter range. With the 68.3% probability distribution of root diameter within a certain root order as a partitioning criterion, the third, fourth, and fifth orders were distributed in the 0.763–1.084-mm, 1.084–1.486-mm, and 1.489–1.858-mm groups, respectively; thus, the main distributions of the diameters of the various root orders did not overlap, indicating that 0.474 mm, 0.753 mm, 1.084 mm, 1.486 mm, and 1.858 mm as threshold values resulted in a one-to-one correlation between diameter intervals and root order; in other words, the ≤0.474 mm, 0.474–0.753-mm, 0.753–1.084-mm, 1.084–1.486-mm, and 1.486–1.858-mm root diameter groups corresponded to first, second, third, fourth, and fifth root orders, respectively. Thus, root diameter interval partitioning contained the largest proportion of root orders.

**Table 2:** Distribution of probability function of root diameter of *Pinus* seedlings at 68.3% distribution interval after Johnson function transformation.

Root order	Mean value	Standard deviation	Quartile of				Transformation formula of Johnson function
			Y_15.85%_	Y_84.15%_	X_15.85%_	X_84.15%_	
I	−0.007	1.018	−1.026	1.012	0.344	0.474	Yi = −3.90828 + 3.62112 * Ln((Xi + 0.502049)/(0.724921 − Xi))
II	0.011	1.008	−0.997	1.020	0.484	0.753	Yi = 2.82472 + 2.52664 * Ln(Xi − 0.263610)
III	0.923	0.160	0.763	1.084	0.763	1.084	\
IV	0.035	0.998	−0.964	1.033	1.084	1.486	Yi = 1.81605 + 1.43391 * Ln((Xi − 0.873319)/(2.54309 − Xi))
V	1.674	0.184	1.489	1.858	1.489	1.858	\

Y_15.85%_ − Y_84.15%_ denotes a threshold value of the 68.3% distribution interval of distribution probability function of the diameter of *Pinus tabuliformis* seedlings after Johnson function transformation, and X_15.85%_ − X_84.15%_ denotes the threshold value inverted from the threshold value of Y_15.85%_ − Y_84.15%_ distribution interval using Johnson function transformation formula corresponding to 68.3% distribution interval of distribution probability function. The same below.

#### Comparison of the four methods

The different methods resulted in different diameter intervals for partitioning roots into classes. The diameter interval division percentages of the four classification methods were calculated and are shown in Table 3.

**Table 3:** Percentages of intervals, as partitioned by four methods, corresponding to each root order of seedlings of *Pinus tabuliformis*.

Diameter intervals (mm)		Percentage of root tips (%)				
		1st order	2nd order	3rd order	4th order	5th order
Probability distribution function interval method	≤0.495	91.2	19.7	0.5		
	0.495–0.777	8.8	67.1	17.3		
	0.777–1.069		11.7	63.1	13.1	
	1.069–1.506		1.5	19.1	72.4	25
	>1.506				14.5	75
Probability distribution function intersection method	≤0.478	84.3	14.8	0.5		
	0.478–0.732	15.7	61.5	2.4		
	0.732–1.062		22.8	75.1	46.2	
	1.062–1.453		0.9	22	17.9	
	>1.453				35.9	100
Normal distribution transformation method	≤0.474	83.2	13.6	0.5		
	0.474–0.753	16.8	70.9	13.4		
	0.753–1.084		14.2	69.7	15.2	
	1.084–1.486		1.3	16.4	68.3	19.8
	>1.486				16.5	80.2
Quartile averaging method	≤0.486	87.9	17.7	0.5		
	0.486–0.768	12.1	68.6	16.4		
	0.768–1.088		12.5	67.2	15.9	
	1.088–1.484		1.2	15.9	67.5	22.5
	>1.484				16.6	77.5

First, based on the proposed evaluation methods, only 67.1% and 63.1% of the second and third orders, respectively, were partitioned by the probability distribution function interval method, thus failing to satisfy the 68.3% criterion. Second, when the probability distribution crossover method was used to divide roots, the percentage of roots contained in the root diameter interval corresponding to second-order roots was only 61.5%. In addition, for fourth-order roots, 46.2% fell in the 0.732–1.062-mm group, 17.9% in the 1.062–1.453-mm group, and 35.9% in the >1.453-mm group, showing considerable overlap with fifth-order roots (100% in the >1.453-mm group), such that they were indistinguishable. Furthermore, the partitioning of fourth-order roots failed to satisfy the 68.3% criterion. Third, the diameter intervals obtained by the quartile averaging method were the closest to those obtained by the normal transformation method, although only 67.2% of third-order roots and 67.5% of fourth-order were accounted for by the quartile averaging method. Thus, this method also failed to meet the 68.3% criterion; however, the partitioning process was simpler and required no data conversion, with simple classification of the original data being sufficient.

Finally, all categories proposed by the normal transformation method satisfied the 68.3% criterion. The most suitable method was determined to be that in which the categories of roots grouped by diameter showed the closest correspondence with root properties other than diameter. The normal transformation method proved superior to the other three in that the diameter intervals corresponded most closely (at least 68.3%) to the morphological characteristics.

### Reasonableness of diameter interval method to describe root morphological characteristics

**Figure 4:**
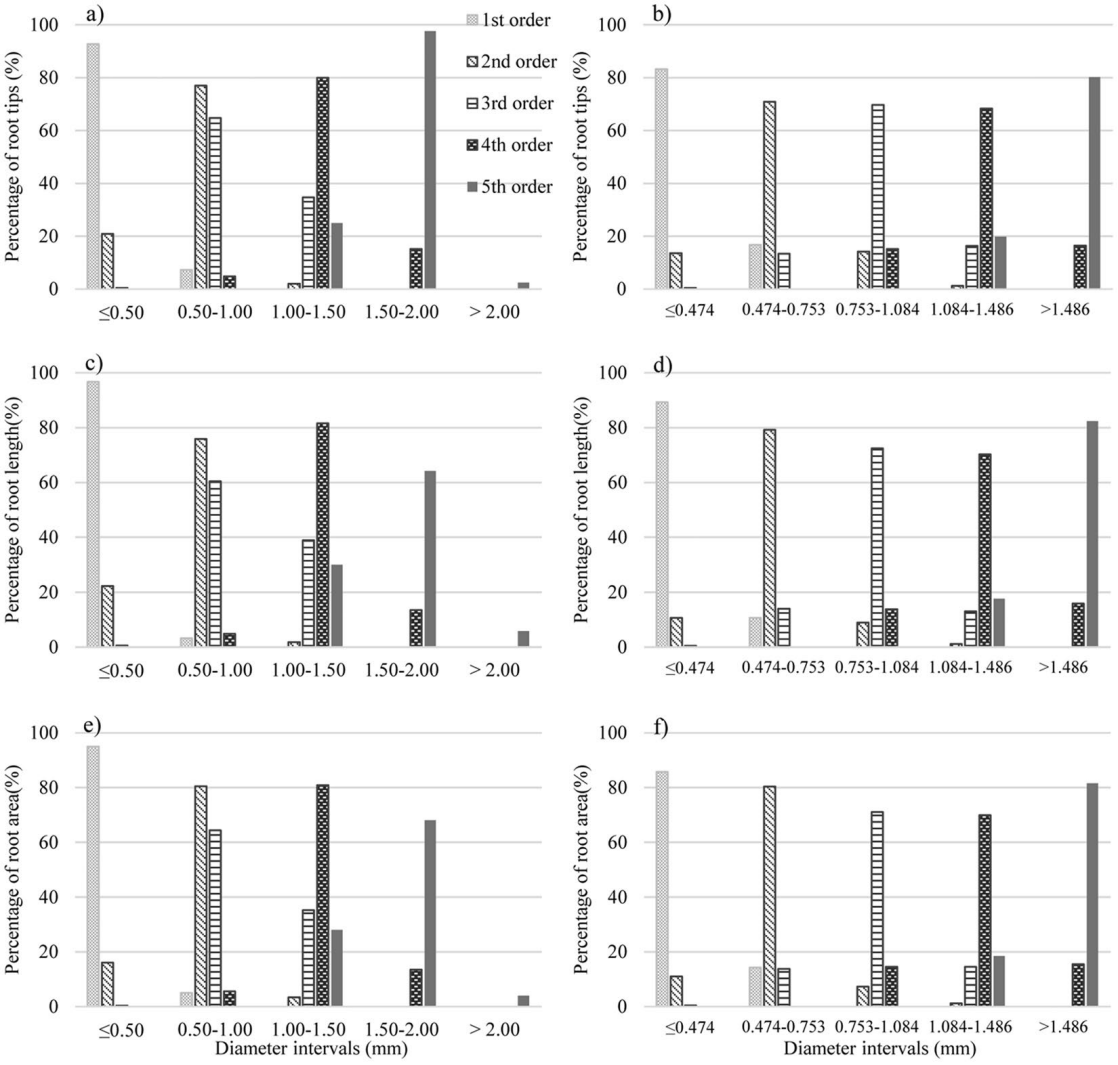
Diameter interval distribution of number of root tips of each root order by (**a**) conventional diameter classification method and (**b**) diameter interval method. Diameter interval distribution of number of root length of each root order by (**c**) conventional diameter classification method and (**d**) diameter interval method. Diameter interval distribution of number of root area of each root order by (**e**) conventional diameter classification method and (**f**) diameter interval method.

To verify that categorizing roots based on diameter rather than order resulted in a close relationship with other morphological characteristics, the normal transformation method was used to obtain diameter intervals (0–0.474 mm, 0.763–1.084 mm, and 1.486–1.858 mm classes). Three characteristics of *P*. *tabuliformis* root morphology, namely, number of root tips, length, and area, in different root orders were re-analysed and compared to the values of the same three indicators in the three categories of roots conventionally grouped by diameter (0–0.5 mm, 0.5–1.0 mm, 1.0–1.5 mm, and 2.0 mm) in terms of percentages of roots falling into each category with reference to each parameter (Fig. 4a,c and e). Conventional grouping failed to reflect the variations due to root order, which was especially evident in the first two groups (0.5–1.0 mm and 1.0–1.5 mm). The 0.5–1.0-mm group contained 64.8% of root tips, 60.5% of root length (RL), and 64.4% of root surface area (RA) of third-order roots; whereas, the 1.0–1.5-mm group contained 34.7%, 38.9%, and 35.3%, respectively, pointing to fragmental partitioning of third-order roots and a failure to reflect the morphologies of the corresponding root-order classes. The 0.5–1.0-mm group contained 77.0% of root tips of second-order roots and 64.8% of root tips of third-order roots, respectively. The same trend was also found for the other indicators, i.e., root length and area (Fig. 4c and e), suggesting that the overlapping distribution failed to distinguish between second- and third-order roots based on morphological features.

In terms of describing root morphology of different roots based on diameter rather than order (Fig. 4b,d and f), the 0–0.474-mm group mainly represented the variation status of first-order roots because the group contained 83.20% of root tips of first-order roots, 89.3% of RL, and 85.7% of RA, whereas the other root orders showed much smaller percentages (below 13.6%) in this group. The 0.474–0.753-mm, 0.753–1.084-mm, 1.084–1.486-mm, and >1.486-mm groups represented 89.3–68.3% of the variation of the second to fifth order roots, respectively.

Linking root morphological characteristics to diameter classes instead of root orders also reduced the differences in the results obtained by these two methods. For example, the total root surface area in the 0.5–1.0-mm group was 31.609 cm², whereas the root surface area of the second-order roots was 17.712 cm², with the difference of 13.897 cm² accounting for 78.5% of the total RA of second-order roots. The total root surface area in the 0.474–0.753-mm group was 19.388 cm², differing by 1.676 cm² from the total RA of the second-order roots, which accounted for 9.0% of the total RA of the second-order roots. This close relationship suggests that the new partitioning method strongly reflected root order when describing root morphology; in other words, diameter and root order gave fairly similar results, reducing the differences in which the two methods reflect root morphology, with the former method being more capable of reflecting the facets of root development.

### Suitability of the diameter interval method to describe root morphological characteristics

The roots of *P*. *bungeana* Zucc. were selected to test whether the diameter interval method was suitable for describing root morphologies. The roots of *P*. *tabuliformis* under a nitrogen enriched environment (simulation of environment changed root morphology) were also used to verify the proposed method.

**Figure 5:**
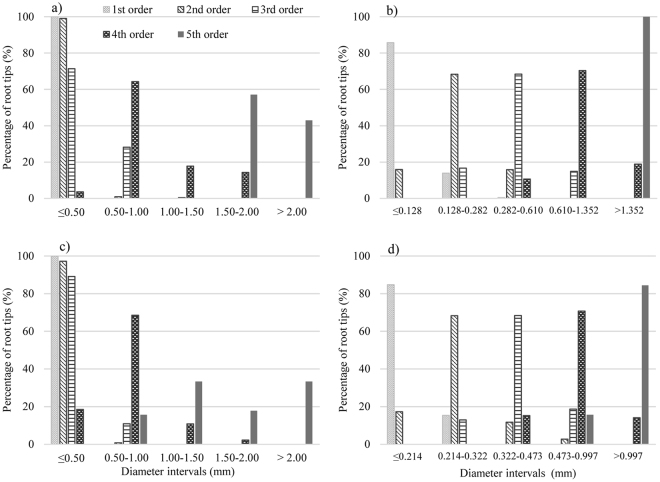
Diameter interval distribution of number of root tips of each root order of *Pinus bungeana Zucc* seedlings by (**a**) conventional diameter classification method and (**b**) diameter interval method. Diameter interval distribution of number of root tips of each root order of *Pinus tabuliformis* grown in nitrogen-enriched soil seedlings by (**c**) conventional diameter classification method and (**d**) diameter interval method.

To ascertain whether the diameter interval method can work with other plants, we collected data on root morphology of seedlings of the biennial *P*. *bungeana* using the normal transformation method (Table 4). The seedlings were grouped by diameter as follows: ≤0.128 mm, 0.128–0.282 mm, 0.282–0.610 mm, 0.610–1.352 mm, and ≥1.352 mm. These root diameter groups contained 85.7%, 68.3%, 68.4%, 70.7%, and 100% of the number of root tips of the first-, second-, third-, fourth- and fifth-order roots, respectively, satisfying the 68.3–95.0–99.7 rule (Fig. 5). Pregitzer *et al*.^10^ pointed out that the diameter of fine roots as a criterion for tree species needs to be made less stringent, and individual differences in species must be given due attention.

**Table 4:** Johnson Distribution of probability function of root diameter of *Pinus bungeana Zucc* seedlings and *P*. *tabuliformis* grown in nitrogen-enriched soil seedlings at 68.3% distribution interval after Johnson function transformation.

Seedlings	Root order	Mean value	Standard deviation	Quantile of Y_15.85%_	Quantile of Y_84.15%_	Quantile of X_15.85%_	Quantile of X_84.15%_	Transformation formula of Johnson function
*Pinus bungeana Zucc* seedlings	I	0.004	0.989	−0.986	0.994	0.094	0.128	Yi = −1.32849 + 0.972258 * ASINH((Xi − 0.0915132)/0.00672982)
	II	−0.004	0.997	−1.001	0.994	0.129	0.282	Yi = 2.22528 + 0.974562 * Ln((Xi − 0.0985883)/(0.945487 − Xi))
	III	0.033	0.994	−0.962	1.027	0.285	0.610	Yi = 1.69500 + 4.07611 * Ln(Xi + 0.237428)
	IV	0.066	0.998	−0.933	1.065	0.634	1.352	Yi = −2.17794 + 1.57702 * Asinh((Xi − 0.419336)/0.243712)
	V	2.001	0.435	1.566	2.436	1.568	2.434	
*P*. *tabuliformis* grown in nitrogen-enriched soil seedlings	I	−0.019	0.983	−1.002	0.965	0.108	0.214	Yi = 1.11652 + 1.46694 * Ln((Xi − 0.0365486)/(0.409780 − Xi))
	II	−0.012	1.005	−1.018	0.993	0.214	0.322	Yi = 3.55139 + 1.28801 * Ln(Xi − 0.185143)
	III	0.398	0.083	0.315	0.481	0.322	0.473	Yi = −2.35500 + 2.22589 * Asinh((Xi − 0.249797)/0.107893)
	IV	−0.033	0.986	−1.020	0.953	0.477	0.997	Yi = 1.60202 + 1.27419 * Ln((Xi − 0.249246)/(2.24633 − Xi))
	V	0.031	1.001	−0.971	1.032	1.009	2.610	Yi = 1.12010 + 0.815060 * Ln((Xi − 0.720331)/(4.73023 − Xi))

Root diameter is also influenced by many environmental factors, including nutrients, water, and temperature^23,35^. In the present study, data on root morphology were also obtained from seedlings of *P*. *tabuliformis* growing in nitrogen-enriched soil. The roots were grouped using conventional grouping, namely <0.5 mm, 0.5–1.0 mm, 1.0–1.5 mm, and 1.5–2.0 mm. The first group accounted for all first-order roots, as well as 97.2% of second-order roots and 89.1% of third-order roots, thus failing to show a one-to-one correspondence between the two grouping methods (i.e., diameter and order). Next, normal transformation was used to repartition the root diameters into 0.214 mm, 0.214–0.322 mm, 0.322–0.473 mm, 0.473–0.997 mm, and >0.997 mm (Table 4). These root diameter groups contained 84.7%, 68.3%, 68.4%, 70.7%, and 84.4% of the number of root tips of the first-, second-, third-, fourth- and fifth-order roots, respectively, satisfying the 68.3–95.0–99.7 rule (Fig. 5) and indicating that, in the context of environmental influence (e.g., adding nitrogen) on fine root diameter, diameter groups can reflect root order morphology.

Chang and Guo studied the relationship between root order and diameter in 45 common tree species in temperate, subtropical, and tropical China^30^; Xu *et al*. studied the morphology of the first five orders of fine roots in four tropical broad-leaved tree species in Hainan Island^12^; and Liu *et al*. studied first-order roots of species used for afforestation^36^, such as *Juglans mandshurica* Maxim., *Phellodendron amurense* Rupr., and *Fraxinus mandschurica* Rupr. These studies all indicated that root diameter varies greatly between tree species, even when roots of the same order are compared and, given such variation in different ecosystems, it is impossible to relate root function to root diameter. In other words, root orders of tree species cannot be linked to root categories based on root diameter (e.g., conventional grouping of roots at diameter intervals of 0.5 mm). This lack of relationship implies that root orders of different plants might correspond to different diameter intervals. Root diameter can be a proxy for root order only if root diameter classes corresponding to root orders of a tree species conform to normal distribution (with or without Johnson function transformation).

## Conclusions

### Advantages and disadvantages of diameter interval method

Using root diameter as a proxy for root order makes sorting a relatively simple process and avoids the need to assign each root to a specific root order. The diameter method provides a new approach for promoting the root order method. However, root morphology varies greatly with species and, for practical application, the relationship between root order and diameter needs to be established separately for individual species. However, such a relationship model may provide a theoretical basis for predicting root characteristics more accurately.

The differences in the order of fine roots and their diameter classes are the key to more accurate determination of the lifespan of fine roots^27^. Wells *et al*. studied the lifespan of fine roots in a *Prunus persica* forest and found that first-order roots grouped by diameter into <0.25 mm, 0.25–0.5 mm, and >0.5 mm categories had lifespans of 74, 121, and 213 d, respectively^3^. The estimated lifespans varied 3-fold, despite the roots being in the same category. Furthermore, root diameter affected the lifespan of fine roots significantly. Root orders reflect the internal heterogeneity of fine roots and can improve our understanding of root function and the accuracy with which other root parameters, such as turnover rates, can be predicted^14,16^. The proposed method for estimating the lifespan of fine roots is simple, and depends on grouping fine roots by diameter. The two methods of grouping roots, namely by diameter and order, can minimize errors in estimating root lifespan when used together.

### Improved method of root sampling

During field sampling, roots are usually damaged or lost to some extent. In the present study, incomplete collection made it difficult to define root order, thereby limiting the application of the root order method in studying fine roots. For example, root loss and failure to distinguish root order in field samples accounted for 3% of overall root weight in a previous study on *P*. *tabuliformis* root morphology influenced by application of nitrogen in the Loess Plateau^23^. It is, therefore, important to estimate the extent of such losses based on statistical analysis of complete or intact roots. Roots can then be grouped by diameter using the proposed method to complete root order data and obtain reliable estimates of the lifespans and turnover rates of fine roots.
